# Treatment patterns and adherence to antihypertensive combination therapies in Japan using a claims database

**DOI:** 10.1038/s41440-018-0127-0

**Published:** 2018-11-16

**Authors:** Takayuki Ishida, Akinori Oh, Shinzo Hiroi, Yukio Shimasaki, Nobuhiro Nishigaki, Takuya Tsuchihashi

**Affiliations:** 1 Takeda Pharmaceutical Company Limited, Japan Medical Affairs, Tokyo, Japan; 2 Steel Memorial Yawata Hospital, Kitakyushu, Japan

**Keywords:** Hypertension, Antihypertensives, Combination therapy, Treatment adherence, Fixed-dose combinations

## Abstract

Fixed-dose combinations (FDCs) for blood pressure control can simplify prescribing, improve medication adherence, and be cost-effective. In Japan, real-world data about the class effects of antihypertensive drugs on medication adherence are limited. Using the nationwide database of medical health claims from Diagnosis Procedure Combination hospitals, treatment patterns and adherence were analyzed for 47,891 patients prescribed antihypertensive medication between April 2014 and March 2015. Adherence was assessed by the proportion of days covered (expressed as % PDC). Patients were prescribed a mean of 2.0 ± 1.0 antihypertensive drugs and 2.4 ± 1.7 pills for their index prescription. Mean adherence overall was 91.5% PDC and was inversely correlated with the number of antihypertensive drugs or pills prescribed on the index date. Mean % PDC was significantly higher (all *P* < 0.0001) for CCB + ARB versus ARB + thiazide diuretic combinations and for CCB + ARB + β-blocker versus CCB + ARB + thiazide diuretic combinations. Adherence was significantly higher (*P* < 0.0001) for FDC (CCB + ARB) versus corresponding single-drug combinations, but not for other comparisons of FDCs versus single-drug combinations. On the other hand, FDCs were not always used effectively; specifically, FDCs were frequently used concomitantly with a single agent(s) from the same drug class(es) as the FDC. From the results of our study, no clear differences were observed in medication adherence according to the presence or absence of FDC formulations, and there were cases in which FDCs were not being utilized effectively to simplify prescribing.

## Introduction

Hypertension is widely recognized as a major risk factor for cardiovascular disease (CVD). Despite the clear downward trend in mean population blood pressure (BP) in Japan over the past 50 years, hypertension remains a significant public health concern [1]. A 2010 survey estimated that there were 43 million hypertensive patients in Japan, with only about 15–30% controlling their BP below 140/90 mmHg [1].

In 2014, the Japanese Society of Hypertension (JSH) issued revised guidelines for the management of hypertension [2]. The 2014 JSH guidelines recognized that combination therapy with different classes of antihypertensive drugs was often needed to achieve and maintain target BP levels. Recommendations for combination therapy are calcium channel blocker (CCB) + angiotensin-converting enzyme (ACE) inhibitor or angiotensin II receptor blocker (ARB); ACE inhibitor or ARB + diuretic; or CCB + diuretic.

High adherence to antihypertensive therapy is associated with lower risks of CVD [3–5], cerebrovascular disease [6], all-cause mortality [5], and hospitalization or emergency department visits [7], as well as improved BP control [8]. A fixed-dose combination (FDC) strategy can simplify prescribing and, as shown in multiple randomized controlled trials of patients at high risk of CVD, improves adherence compared with a multidrug strategy [9–13]. A FDC strategy was also shown to be cost-effective for prevention of fatal and nonfatal cardiovascular events [14]. The 2014 JSH guidelines support the concept of prescribing FDC antihypertensive drugs to improve medication adherence and control BP [2].

At present, there are relatively limited real-world data from nationwide databases about the class effects of antihypertensive drugs on medication adherence in Japan. Reports have been limited to randomized clinical trials [8, 15] or experience in a single hypertension clinic [16], which is inadequate to describe usage on a national scale. A recent analysis of real-world Japanese antihypertensive treatment was restricted to second- and third-line treatment after initial ARB therapy [17]. Unlike clinical research where drug use is strictly controlled, drug use in everyday clinical practice is varied. By using a claim-based database that reflects actual clinical practice, we speculated that treatment adherence rates would differ from those reported in previous clinical research. The compositions and dosages of currently available FDC antihypertensives are limited, and the pervasiveness of FDC is everyday practice is low, often resulting in ineffective use of these drugs. We hypothesized that FDC antihypertensive drugs currently make only a small contribution to medication adherence.

In the current study, treatment patterns and medication adherence to a range of antihypertensive drug classes, including some common two-drug-class and three-drug-class combinations, were analyzed. We also examined whether FDC antihypertensive drugs were being prescribed on their own or concomitantly with other antihypertensive drugs.

## Methods

A retrospective analysis was performed using clinical data from the Diagnosis Procedure Combination (DPC) database, which was developed by the Medical Data Vision (MDV) Company (Tokyo, Japan). DPC is a nationwide database of anonymized medical health claims and administrative data covering individuals treated as inpatients or outpatients at hospitals in Japan participating in the DPC payment system [18, 19]. The database stores information on diagnoses, medical costs, prescriptions (including prescription dates) and, for some hospitals, blood test results.

Subjects were registered in DPC hospitals during the target selection period of April 2014 to March 2015. Data for each patient were collected for the look-back period (12 months before the date of first prescription or “index date”) and follow-up period (12 months after the index date). Data were extracted for patients meeting the selection criteria (defined below).

### Selection and exclusion criteria

**Fig. 1:**
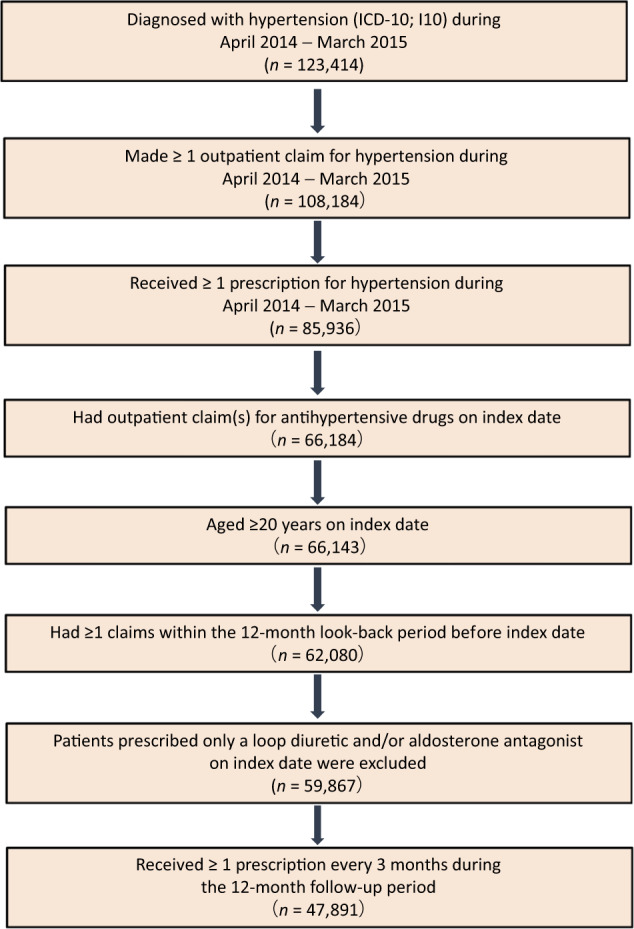
Patient disposition

Selection and exclusion criteria are presented in Fig. 1. Patients diagnosed with essential (primary) hypertension (ICD-10; I10) were eligible for inclusion in the study. Selected patients were those with one or more outpatient claims for hypertension; received at least one antihypertensive prescription as an outpatient between April 2014 and March 2015 (index period); were aged 20 years or older on the index date; had outpatient claim(s) for antihypertensive drugs on the index date; had made one or more claims within the 12-month look-back period; and received at least one prescription every 3 months during the index period. Patients who were prescribed solely a loop diuretic and/or aldosterone antagonist on the index date were excluded, as these agents have indications apart from hypertension.

### Outcomes

Medication adherence was assessed by proportion of days covered (PDC), which was calculated according to the formula:

Total number of prescription days covered for defined drugs of interest/Total number of days in the follow-up period.

Adherence at 12 months is expressed as % PDC and was categorized as high (PDC ≥ 80%), intermediate (PDC 40–79%), or low (PDC ≤ 39%) [20].

### Ethical considerations

The MDV database used for the study stored anonymized medical data that had been encrypted before entry. According to Ethical Guidelines for Epidemiological Research issued by the Japanese Ministry of Health, Welfare and Labour, ethical approval and informed consent were not applicable. Data were analyzed by IQVIA Solutions Japan K.K. and stored in password-protected stand-alone personal computers not connected to a local area network.

### Statistical analysis

Descriptive statistics were used to summarize patient demographics, baseline comorbidities, and number of prescribed antihypertensive drugs. Continuous data are presented as the mean ± standard deviation (s.d.), and categorical data are expressed as number and percentage of patients. Prescription rates for each class of antihypertensive drug combination are presented as percentages for the overall population and for each category of PDC-based adherence. Medication adherence rates for classes of antihypertensive drugs were compared statistically using the *t*-test. Analyses were performed using SAS^®^ Version 9.2 (SAS Institute Inc., Cary, NC, USA).

## Results

Baseline demographics and clinical characteristics of the overall population and by PDC category (high ≥ 80%; intermediate 40–49%; low ≤ 39%) are summarized in Table 1. Included patients (*n* = 47,891) were 57.0% male and had a mean age of 70.1 years; 93.5% of patients (*n* = 44,759) had been treated with antihypertensive drugs in the look-back period. The most common comorbidity was dyslipidemia (59.6%), followed by hospitalized heart disease (53.2%), and diabetes (26.1%). Most patients (*n* = 42,352; 88.4% of total) were in the high-medication-adherence category.

**Table 1:** Demographics and clinical characteristics of the overall population and of patients stratified according to their level of medication adherence

Patient characteristics	Overall population (*n* = 47,891)	Proportion of days covered (PDC)		
		High ( ≥ 80%) (*n* = 42,352)	Intermediate (40–79%) (*n* = 3124)	Low ( ≤ 39%) (*n* = 2415)
Age, years	70.1 ± 11.5	70.0 ± 11.4	69.6 ± 12.4	71.3 ± 11.7
Gender, male	27,293 (57.0)	24,061 (56.8)	1819 (58.2)	1413 (58.5)
Comorbidities				
Diabetes	12,508 (26.1)	11,075 (26.2)	792 (25.4)	641 (26.5)
Dyslipidemia	28,538 (59.6)	25,399 (60.0)	1716 (54.9)	1423 (58.9)
Gout/hyperuricemia	9026 (18.9)	7764 (18.3)	677 (21.7)	585 (24.2)
Renal diseases	4803 (10.0)	3890 (9.2)	463 (14.8)	450 (18.6)
Hospitalized heart disease	25,487 (53.2)	22,224 (52.5)	1704 (54.6)	1559 (64.6)
Hospitalized cerebrovascular disease	9663 (20.2)	8633 (20.4)	562 (18.0)	468 (19.4)
Hospitalized patients	8245 (17.2)	6434 (15.2)	1011 (32.4)	800 (33.1)
Antihypertensive drugs prescribed on index date	2.0 ± 1.0	1.9 ± 1.0	2.2 ± 1.0	2.7 ± 1.1
Antihypertensive pills prescribed on index date	2.4 ± 1.7	2.3 ± 1.6	2.7 ± 1.9	3.3 ± 2.0
Oral drugs prescribed for lifestyle-related^a^ disease treatment on index date	3.6 ± 2.0	3.6 ± 2.0	3.8 ± 2.2	4.4 ± 2.2
Pills prescribed for lifestyle-related^a^ disease treatment on index date	4.8 ± 3.5	4.7 ± 3.5	5.1 ± 3.6	5.9 ± 3.8
Diabetes				
Oral drugs for diabetes prescribed on index date	0.44 ± 0.92	0.44 ± 0.93	0.41 ± 0.88	0.39 ± 0.83
Pills for diabetes prescribed on index date	0.84 ± 1.99	0.85 ± 2.01	0.76 ± 1.83	0.73 ± 1.77
Dyslipidemia				
Oral drugs for dyslipidemia prescribed on index date	0.54 ± 0.61	0.54 ± 0.61	0.49 ± 0.61	0.53 ± 0.61
Pills for dyslipidemia prescribed on index date	0.65 ± 0.91	0.66 ± 0.91	0.60 ± 0.90	0.65 ± 0.90
Gout/hyperuricemia				
Oral drugs for gout/hyperuricemia prescribed on index date	0.15 ± 0.36	0.14 ± 0.35	0.18 ± 0.39	0.20 ± 0.40
Pills for gout/hyperuricemia prescribed on index date	0.17 ± 0.45	0.17 ± 0.44	0.20 ± 0.47	0.23 ± 0.48
Antithrombotic agents				
Oral antithrombotic drugs prescribed on index date	0.51 ± 0.72	0.50 ± 0.71	0.51 ± 0.73	0.63 ± 0.78
Antithrombotic pills prescribed on index date	0.79 ± 1.32	0.78 ± 1.31	0.79 ± 1.32	0.98 ± 1.43

Data are expressed as mean ± s.d. or number (%) of patients

^a^Lifestyle-related drugs included antihypertensive, antidiabetic, antidyslipidemic, antigout/hyperuricemic, and antithrombotic drugs

Overall, patients were prescribed a mean of 2.0 ± 1.0 antihypertensive drugs and 2.4 ± 1.7 antihypertensive pills on the index date (Table 1). The mean number of oral drugs and pills (including antihypertensive, antidiabetic, antidyslipidemic, antigout/hyperuricemic, and antithrombotic drugs) prescribed for lifestyle-related diseases on the index date was 3.6 ± 2.0 and 4.8 ± 3.5, respectively.

The mean number of antihypertensive drugs and antihypertensive pills prescribed on the index date increased inversely with adherence, from 1.9 ± 1.0 drugs and 2.3 ± 1.6 pills in patients with high medication adherence to 2.7 ± 1.1 drugs and 3.3 ± 2.0 pills in patients with low adherence (Table 1). Corresponding numbers for lifestyle-related disease treatments were 3.6 ± 2.0 oral drugs and 4.7 ± 3.5 pills for patients with high adherence and 4.4 ± 2.2 oral drugs and 5.9 ± 3.8 pills for patients with low adherence.

For analyses of medication adherence and treatment patterns, the main antihypertensive combinations of interest were the two-drug-class combinations of CCB + ARB and ARB + thiazide diuretics and the three-drug-class combinations of CCB + ARB + β-blocker and CCB + ARB + thiazide diuretics. FDC formulations were available for CCB + ARB and ARB + thiazide diuretic combinations.

### Medication adherence

**Fig. 2:**
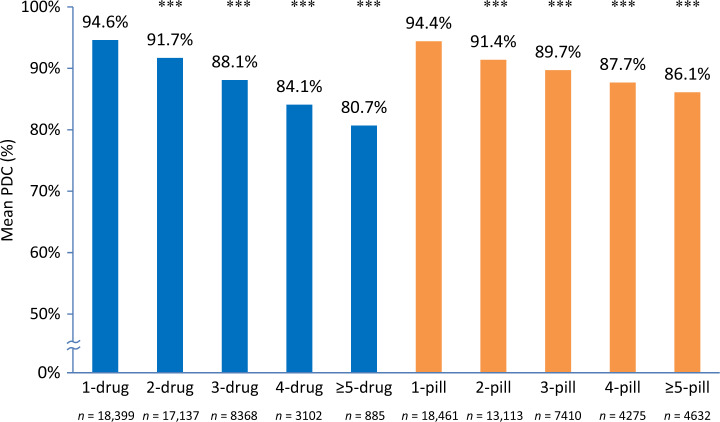
Treatment adherence: mean % proportion of days covered (PDC) according to the number of antihypertensive drugs and pills prescribed on the index date. ***P < 0.0001 for comparisons between 1-drug and 2-, 3-, 4-, and ≥ 5-drug regimens and between 1-pill and 2-, 3-, 4-, and ≥ 5-pill regimens

Mean adherence (% PDC) to antihypertensive medication in the overall population was 91.5 ± 0.2%. Mean % PDC was inversely correlated with the number of oral antihypertensive drugs and pills prescribed on the index date, ranging from 94.6% with 1 drug to 80.7% with ≥ 5 drugs and from 94.4% with 1 pill to 86.1% with ≥ 5 pills. Comparisons of mean % PDC were statistically significant (*P* < 0.0001 for all comparisons) between 1 drug and 2, 3, 4, and ≥ 5 drugs per day and between 1 pill and 2, 3, 4, and ≥ 5 pills per day (Fig. 2).

Adherence to antihypertensive monotherapy was high overall (PDC ≥ 80%) and varied according to drug class. Mean % PDC was 95.0% for CCBs (*n* = 8702); 95.2% for ARBs (*n* = 6958); 93.5% for β-blockers (*n* = 1733); 90.4% for ACE inhibitors (*n* = 726); 89.0% for α-blockers (*n* = 181); and 87.0% for thiazide diuretics (*n* = 87). Compared with ARBs (the drug class with highest adherence), mean % PDC was statistically significantly lower for β-blockers (*P* < 0.0001), ACE inhibitors (*P* < 0.0001), α-blockers (*P* = 0.0002), and thiazide diuretics (*P* = 0.0016), but not for CCBs (*P* = 0.256).

**Fig. 3:**
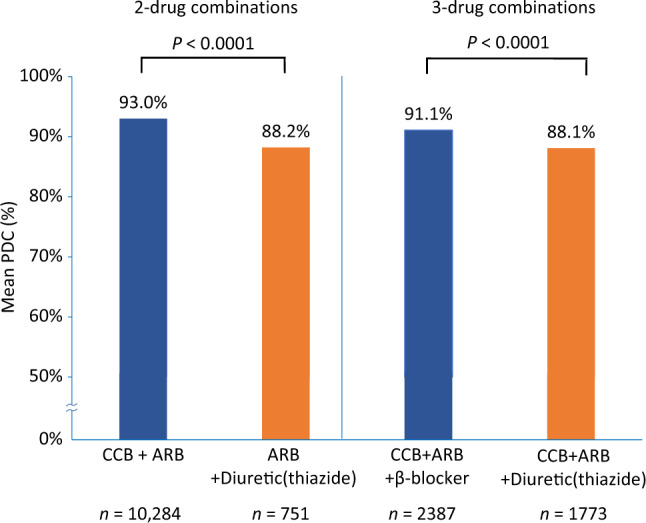
Treatment adherence: mean % proportion of days covered (PDC) for antihypertensive drug combinations of interest

Comparison of adherence rates for two-drug-class and three-drug-class antihypertensive therapy combinations showed that mean % PDC was significantly higher for CCB + ARB combinations than for ARB + thiazide diuretic combinations (*P* < 0.0001) and was significantly higher for CCB + ARB + β-blocker combinations than for CCB + ARB + thiazide diuretic combinations (*P* < 0.0001) (Fig. 3).

**Fig. 4:**
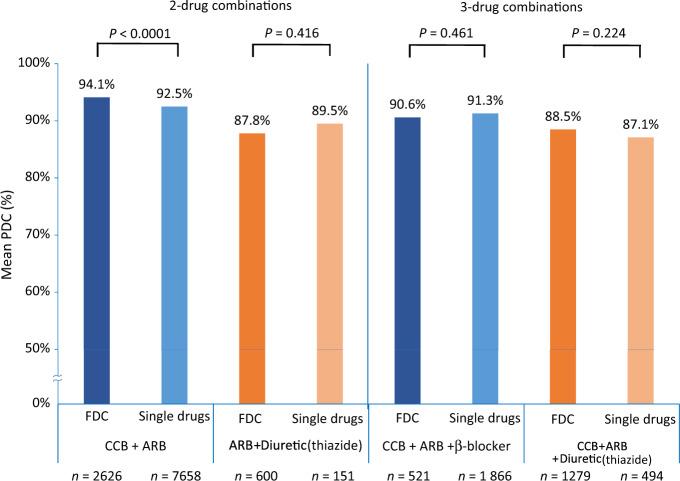
Treatment adherence: mean % proportion of days covered (PDC) for antihypertensive drug combinations of interest, prescribed as fixed-dose combinations (FDCs) with or without a single agent(s) of the same drug class(es) as the FDC, or as combinations of corresponding single drugs

Adherence was high (PDC ≥ 80%) for all two-drug-class and three-drug-class antihypertensive therapy combinations, prescribed as FDCs or as corresponding single-drug combinations. The FDC category encompassed all cases in which a FDC was part of the index prescription, including instances where a single agent(s) of the same antihypertensive drug class(es) as the FDC was (were) prescribed concomitantly. Adherence rates were significantly higher for FDC CCB + ARB than for single-drug combinations (*P* < 0.0001), whereas adherence rates for FDC ARB + thiazide diuretic, FDC CCB + ARB + β-blocker, and FDC CCB + ARB + thiazide diuretic did not differ significantly from those for corresponding single-drug combinations (Fig. 4).

### Treatment patterns for FDC

In instances where a FDC was part of the index prescription, the frequency of prescriptions for a FDC plus a concomitant single agent(s) of the same drug class(es) as the FDC was 3.7% with ARB + thiazide diuretic combinations, 7.1% with CCB + ARB + thiazide diuretic combinations, 14.9% with CCB + ARB combinations, and 25.3% with CCB + ARB + β-blocker combinations.

## Discussion

In this study, treatment patterns and adherence were analyzed in approximately 48,000 patients who were prescribed antihypertensive drugs during the target selection period of April 2014 to March 2015, using the DPC clinical database of anonymized medical health claims in Japan. Adherence was evaluated in several ways: according to the number of oral antihypertensive drugs and pills prescribed on the index date; according to the type of two-drug-class or three-drug-class antihypertensive regimen prescribed on the index date; and according to whether the index prescription was for a FDC (± concomitant prescription of a single agent[s] of the same drug class[es] as the FDC) or for single drugs in combination.

Overall, approximately 88% of patients had a high level of adherence (PDC ≥ 80%) to their prescribed antihypertensive medication. This finding is consistent with the results of an adherence study conducted in routine clinical practice which reported that adherence to antihypertensive drug therapy was considerably higher than that to antidiabetic and antidyslipidemic drugs [21].

Regarding population characteristics and adherence, the hospitalization rate was twofold higher in the intermediate- and low-adherence subgroups compared with the high-adherence subgroup. It is not uncommon for hospitalized patients to have several complications resulting in the need for many different medicines. Moreover, following discharge, drug switching may occur, which can contribute to reduced adherence. On the index date, the group with the lowest level of adherence to antihypertensive drug therapy had been prescribed a mean of 5.9 pills of medication for lifestyle-related diseases. This finding aligns with a large systematic review indicating that compliance is negatively correlated with the number of daily doses [22].

We also observed a higher prevalence of renal diseases in the intermediate- or low-adherence subgroups compared with the high-adherence subgroup. A study from Taiwan that also used health insurance data to assess use of FDC and single-drug combinations of ARB and thiazide diuretics in newly-diagnosed hypertensive patients found a tendency for patients with renal diseases or depression to have poor adherence [23].

We found that adherence to CCB + ARB combinations was higher than that to ARB + thiazide diuretic combinations and, among three-drug-class combinations, that adherence was higher to CCB + ARB + β-blocker combinations than that to CCB + ARB + thiazide diuretic combinations. Side effects of drugs are a major factor of poor adherence [24]. Diuretics, for example, can cause urinary frequency, erectile dysfunction, fatigue, and muscle cramps, which may be intolerable to patients [25]. Diuretics can also induce metabolic and electrolyte abnormalities, which may lead to their discontinuation by physicians [26].

Comparisons between FDCs and corresponding single-drug combinations showed that adherence was significantly better (*P* < 0.0001) only with FDC CCB + ARB; no other comparisons between FDCs and single-drug combinations were statistically significant. The above-mentioned Taiwanese study using a health insurance database found that adherence and persistence rates were significantly higher with FDC ARB + thiazide diuretic compared with corresponding single-drug combinations [23]. Previous studies using healthcare or insurance databases reported low rates of adherence and persistence with diuretic monotherapy for treatment of hypertension [27, 28], which were improved by using diuretic-containing FDCs [28]. This contrasts with our results and may be attributable to different analysis conditions and/or a lack of statistical power, as only 751 patients in our analysis received an ARB + thiazide diuretic combination as their index prescription (FDC: *n* = 600; single-drug combinations: *n* = 151), whereas >10,000 patients had been prescribed a CCB + ARB combination. A 2010 meta-analysis of three cohort studies and two clinical trials reporting on drug compliance (*n* = 17,999) concluded that FDC use was associated with significantly better adherence compared with use of corresponding single-drug combinations (odds ratio: 1.21; 95% confidence interval: 1.03–1.43; *P* = 0.02) [29]. In more recent analyses, superior adherence to FDC antihypertensive drug therapy was demonstrated for FDC CCB + ARB [30] and for FDC CCB + ARB + thiazide diuretic [31, 32]. Conversely, a randomized controlled trial of ARB + thiazide diuretic therapy failed to demonstrate improved adherence to FDCs compared with corresponding single-drug combinations [15].

For index prescriptions of CCB + ARB combinations, approximately 25% of patients were prescribed a FDC, whereas for index prescriptions of ARB + thiazide diuretic combinations, this figure rose to approximately 80%. A major factor influencing the decision to prescribe a FDC ARB + thiazide diuretic may be the low dose of diuretic, which is equivalent to half or even a quarter of a regular pill. Regarding the use of FDC plus concomitant single agent(s) from the same drug class(es) as the FDC, prescribing rates were lowest with ARB + thiazide diuretic combinations (3.7%) and highest with CCB + ARB combinations (14.9%). A limitation of the DPC database is that the timing of medication administration cannot be analyzed. Thus, the prescribing patterns we observed with FDCs may be attributed to dosage and/or timing adjustments (i.e., chronotherapy). Use of FDC formulations instead of single-drug combinations may be useful in terms of reducing prescription costs [33], correcting night-time hypertension, and limiting adverse events. However, the possibility exists that FDC formulations are not being utilized effectively in terms of simplifying prescribing.

Some limitations of the study relate to the characteristics of the database. Not all patients in DPC hospitals can be tracked accurately, initial diagnoses (i.e., essential hypertension) may have differed from final diagnoses, data were lacking about residual medication, and data were insufficient with regard to clinical laboratory values including BP readings. As mentioned previously, medication dosages and timing of administration were not studied. Chronotherapy may prove to be an important consideration for effective BP control [34]. Some patients received FDC plus single agent(s) of the same drug class(es) as the FDC, which complicated data interpretation. It is conceivable that a proportion of patients were prescribed antihypertensive drugs for heart disease rather than for BP control. Finally, the number of patients varied considerably between groups, with lower numbers limiting the statistical power of some analyses. Other study limitations related to the method used to estimate medication adherence. Although, for claims-based studies, PDC is preferred over the medication possession ratio to estimate adherence because it prospectively adjusts for overlapping days supplied, we may have overestimated actual adherence by overlooking leftover medications or the prescribing of drugs to patients with leftover medications. Finally, since outpatients and inpatients were managed using different types of medication, differences in the hospitalization rate between in- and outpatient groups may have had an influence on treatment adherence.

In conclusion, analyses using a Japanese claims database indicated that poor adherence to antihypertensive treatment was associated with a higher number of drugs and a higher number of pills prescribed. Among common two-drug- and three-drug-combination regimens, medication adherence was significantly higher in patients receiving nondiuretic-containing than diuretic-containing combinations. There was no clear difference in medication adherence to FDC and corresponding single-drug combinations. We found that FDCs were not always used effectively; specifically, FDCs were frequently used concomitantly with single agent(s) from the same drug class(es) as the FDC. The benefits FDCs offer to patients include greater convenience through simplified prescribing and reduced burden in terms of medication costs. Effective use of FDCs may also help to improve treatment adherence. The study findings suggest the need to identify measures to facilitate more effective use of FDCs.

## References

[1] Miura K, Nagai M, Ohkubo T (2013). Epidemiology of hypertension in Japan: where are we now?. Circ J.

[2] Shimamoto K, Ando K, Fujita T, Hasebe N, Higaki J, Horiuchi M (2014). Japanese Society of Hypertension Committee for Guidelines for the Management of Hypertension. The Japanese Society of Hypertension Guidelines for the Management of Hypertension (JSH 2014). Hypertens Res.

[3] Perreault S, Dragomir A, White M, Lalonde L, Blais L, Bérard A (2009). Better adherence to antihypertensive agents and risk reduction of chronic heart failure. J Intern Med.

[4] Corrao G, Parodi A, Nicotra F, Zambon A, Merlino L, Cesana G (2011). Better compliance to antihypertensive medications reduces cardiovascular risk. J Hypertens.

[5] Shin S, Song H, Oh SK, Choi KE, Kim H, Jang S (2013). Effect of antihypertensive medication adherence on hospitalization for cardiovascular disease and mortality in hypertensive patients. Hypertens Res.

[6] Kettani FZ, Dragomir A, Côté R, Roy L, Bérard A, Blais L (2009). Impact of a better adherence to antihypertensive agents on cerebrovascular disease for primary prevention. Stroke.

[7] Juarez DT, Tan C, Davis J, Mau M (2013). Factors affecting sustained medication adherence and its impact on health care utilization in patients with diabetes. J Pharm Health Serv Res.

[8] Matsumura K, Arima H, Tominaga M, Ohtsubo T, Sasaguri T, Fujii K.et al.Comfort Investigators. Impact of antihypertensive medication adherence on blood pressure control in hypertension: the COMFORT study. QJM. 2013;106:909–14.. 10.1093/qjmed/hct121 23696676

[9] Thom S, Poulter N, Field J, Patel A, Prabhakaran D, Stanton A.et al.UMPIRE Collaborative Group. Effects of a fixed-dose combination strategy on adherence and risk factors in patients with or at high risk of CVD: the UMPIRE randomized clinical trial. JAMA. 2013;310:918–29.. 10.1001/jama.2013.277064 24002278

[10] Selak V, Elley CR, Bullen C, Crengle S, Wadham A, Rafter N (2014). Effect of fixed dose combination treatment on adherence and risk factor control among patients at high risk of cardiovascular disease: randomised controlled trial in primary care. BMJ.

[11] Patel A, Cass A, Peiris D, Usherwood T, Brown A, Jan S.et al.Kanyini Guidelines Adherence with the Polypill (Kanyini GAP) Collaboration. A pragmatic randomized trial of a polypill-based strategy to improve use of indicated preventive treatments in people at high cardiovascular disease risk. Eur J Prev Cardiol. 2015;22:920–30.. 10.1177/2047487314530382 24676715

[12] Lafeber M, Grobbee DE, Schrover IM, Thom S, Webster R, Rodgers A (2015). Comparison of a morning polypill, evening polypill and individual pills on LDL-cholesterol, ambulatory blood pressure and adherence in high-risk patients; a randomized crossover trial. Int J Cardiol.

[13] Bahiru E, de Cates AN, Farr MR, Jarvis MC, Palla M, Rees K (2017). Fixed-dose combination therapy for the prevention of atherosclerotic cardiovascular diseases. Cochrane Database Syst Rev.

[14] Becerra V, Gracia A, Desai K, Abogunrin S, Brand S, Chapman R (2015). Cost-effectiveness and public health benefit of secondary cardiovascular disease prevention from improved adherence using a polypill in the UK. BMJ Open.

[15] Matsumura K, Arima H, Tominaga M, Ohtsubo T, Sasaguri T, Fujii K (2012). Does a combination pill of antihypertensive drugs improve medication adherence in Japanese? A randomized controlled trial. Circ J.

[16] Kansui Y, Ibaraki A, Goto K, Haga Y, Seki T, Takiguchi T (2016). Trends in blood pressure control and medication use during 20 years in a hypertension clinic in Japan. Clin Exp Hypertens.

[17] Hiroi S, Shimasaki Y, Kikuchi T, Otsuka Y, Iwasaki K, Ohishi M (2016). Analysis of second- and third-line antihypertensive treatments after initial therapy with an angiotensin II receptor blocker using real-world Japanese data. Hypertens Res.

[18] Tanaka S, Seto K, Kawakami K (2015). Pharmacoepidemiology in Japan: medical databases and research achievements. J Pharm Health Care Sci.

[19] Ishikawa KB (2016). Medical big data for research use: current status and related issues. Jpn Med Assoc J.

[20] Levi M, Pasqua A, Cricelli I, Cricelli C, Piccinni C, Parretti D (2016). Patient adherence to olmesartan/amlodipine combinations: fixed versus extemporaneous combinations. J Manag Care Spec Pharm.

[21] Colombo GL, Agabiti-Rosei E, Margonato A, Mencacci C, Montecucco CM, Trevisan R (2016). Impact of substitution among generic drugs on persistence and adherence: a retrospective claims data study from 2 local healthcare units in the Lombardy Region of Italy. Atheroscler Suppl.

[22] Claxton AJ, Cramer J, Pierce C (2001). A systematic review of the associations between dose regimens and medication compliance. Clin Ther.

[23] Hsu CI, Hsiao FY, Wu FL, Shen LJ (2015). Adherence and medication utilisation patterns of fixed-dose and free combination of angiotensin receptor blocker/thiazide diuretics among newly diagnosed hypertensive patients: a population-based cohort study. Int J Clin Pract.

[24] Osterberg L, Blaschke T (2005). Adherence to medication. N Engl J Med.

[25] Kronish IM, Woodward M, Sergie Z, Ogedegbe G, Falzon L, Mann DM (2011). Meta-analysis: impact of drug class on adherence to antihypertensives. Circulation.

[26] Tani S, Asayama K, Oiwa K, Harasawa S, Okubo K, Takahashi A (2017). The effects of increasing calcium channel blocker dose vs. adding a diuretic to treatment regimens for patients with uncontrolled hypertension. Hypertens Res.

[27] Mancia G, Zambon A, Soranna D, Merlino L, Corrao G (2014). Factors involved in the discontinuation of antihypertensive drug therapy: an analysis from real life data. J Hypertens.

[28] Schulz M, Krueger K, Schuessel K, Friedland K, Laufs U, Mueller WE (2016). Medication adherence and persistence according to different antihypertensive drug classes: a retrospective cohort study of 255,500 patients. Int J Cardiol.

[29] Gupta AK, Arshad S, Poulter NR (2010). Compliance, safety, and effectiveness of fixed-dose combinations of antihypertensive agents: a meta-analysis. Hypertension.

[30] Levi M, Pasqua A, Cricelli I, Cricelli C, Piccinni C, Parretti D (2016). Patient adherence to olmesartan/amlodipine combinations: fixed versus extemporaneous combinations. patient adherence to olmesartan/amlodipine combinations: fixed versus extemporaneous combinations. J Manag Care Spec Pharm.

[31] Bramlage P, Ketelhut R, Fronk EM, Wolf WP, Smolnik R, Zemmrich C (2014). Clinical impact of patient adherence to a fixed-dose combination of olmesartan, amlodipine and hydrochlorothiazide. Clin Drug Investig.

[32] Machnicki G, Ong SH, Chen W, Wei ZJ, Kahler KH (2015). Comparison of amlodipine/valsartan/hydrochlorothiazide single pill combination and free combination: adherence, persistence, healthcare utilization and costs. Curr Med Res Opin.

[33] Taylor AA, Shoheiber O (2003). Adherence to antihypertensive therapy with fixed-dose amlodipine besylate/benazepril HCl versus comparable component-based therapy. Congest Heart Fail.

[34] Hermida RC, Ayala DE, Smolensky MH, Fernández JR, Mojón A, Portaluppi F (2016). Chronotherapy with conventional blood pressure medications improves management of hypertension and reduces cardiovascular and stroke risks. Hypertens Res.

